# Intracellular Mono-ADP-Ribosylation in Signaling and Disease

**DOI:** 10.3390/cells4040569

**Published:** 2015-09-25

**Authors:** Mareike Bütepage, Laura Eckei, Patricia Verheugd, Bernhard Lüscher

**Affiliations:** Institute of Biochemistry and Molecular Biology, Medical School, RWTH Aachen University, Pauwelsstrasse 30, 52057 Aachen, Germany; E-Mails: mbuetepage@ukaachen.de (M.B.); leckei@ukaachen.de (L.E.)

**Keywords:** ADP-ribosylation, cancer, DNA repair, endoplasmic reticulum, gene transcription, immunity, infection, NF-κB, signaling, stress

## Abstract

A key process in the regulation of protein activities and thus cellular signaling pathways is the modification of proteins by post-translational mechanisms. Knowledge about the enzymes (writers and erasers) that attach and remove post-translational modifications, the targets that are modified and the functional consequences elicited by specific modifications, is crucial for understanding cell biological processes. Moreover detailed knowledge about these mechanisms and pathways helps to elucidate the molecular causes of various diseases and in defining potential targets for therapeutic approaches. Intracellular adenosine diphosphate (ADP)-ribosylation refers to the nicotinamide adenine dinucleotide (NAD^+^)-dependent modification of proteins with ADP-ribose and is catalyzed by enzymes of the ARTD (ADP-ribosyltransferase diphtheria toxin like, also known as PARP) family as well as some members of the Sirtuin family. Poly-ADP-ribosylation is relatively well understood with inhibitors being used as anti-cancer agents. However, the majority of ARTD enzymes and the ADP-ribosylating Sirtuins are restricted to catalyzing mono-ADP-ribosylation. Although writers, readers and erasers of intracellular mono-ADP-ribosylation have been identified only recently, it is becoming more and more evident that this reversible post-translational modification is capable of modulating key intracellular processes and signaling pathways. These include signal transduction mechanisms, stress pathways associated with the endoplasmic reticulum and stress granules, and chromatin-associated processes such as transcription and DNA repair. We hypothesize that mono-ADP-ribosylation controls, through these different pathways, the development of cancer and infectious diseases.

## 1. Introduction 

Adenosine diphosphate (ADP)-ribosylation is a reversible post-translational modification (PTM) of proteins that is catalyzed by ADP-ribosyltransferases (ARTs) and certain members of the Sirtuin family [1,2,3]. In ADP-ribosylation reactions, the cofactor nicotinamide adenine dinucleotide (NAD^+^) is used to covalently attach residues of ADP-ribose to specific acceptor amino acids of substrate proteins with release of nicotinamide. ARTs share a structurally conserved catalytic domain. Based on the homology of these catalytic domains either to cholera or to diphtheria toxin, mammalian ARTs are categorized as members of the ADP-ribosyltransferase cholera toxin-like (ARTCs) or diphtheria toxin-like (ARTDs) sub-families, respectively. ARTC enzymes are GPI-anchored extracellular or secreted enzymes that transfer single ADP-ribose residues to their substrates [4,5]. In contrast, ARTDs are intracellular enzymes able to transfer either a single ADP-ribose residue to an acceptor amino acid, a process referred to as mono-ADP-ribosylation (MARylation), or they are able to attach additional ADP-ribose residues with ADP-ribose being the acceptor, resulting in either linear or branched chains of ADP-ribose (poly-ADP-ribosylation or PARylation) [6]. The founding member of the ARTD family, ARTD1, was first shown to PARylate itself and substrate proteins, which led to the initial designation PARP1 for poly-ADP-ribose polymerase 1. The family of proteins with related catalytic domains was therefore termed PARP family. However, after the discovery of sub-family members being capable of only MARylation, a new nomenclature and classification has been proposed that we shall use here [7] (Table 1). The ability of intracellular ART enzymes to form ADP-ribose polymers depends on the defined H-Y-E amino acid signature in the catalytic center. Enzymes with this signature transfer multiple ADP-ribose units in an iterative process to their substrates using the glutamate to activate NAD^+^. The catalytic glutamate residue is replaced in enzymes of the mono-ARTD group (H-Y-E variants). These enzymes instead employ a glutamate residue of the substrate for the transfer of ADP-ribose (substrate-assisted catalysis), which when modified is then no longer available for the activation of additional NAD^+^ cofactor molecules [6]. Therefore, besides ARTD1, ARTD2 (PARP2), ARTD5 (PARP5A, Tankyrase 1) and ARTD6 (Tankyrase 2) synthesize poly- or oligo-ADP-ribose chains, whereas ARTD7/8 (PARP15, PARP14), ARTD10–12 (PARP10–12), and ARTD14–17 (PARP8, -7, -16, -6) are classified as mono-ADP-ribosyltransferases (mono-ARTDs) [6,7]. Additional amino acid substitutions as in ARTD9 and ARTD13 (PARP9, -13) prohibit NAD^+^-binding and render these proteins catalytically inactive [6,8]. Although ARTD3 and ARTD4 (PARP3, -4) were initially proposed to have PARylation activity based on their classical H-Y-E signature [6], MARylation activity for both enzymes has been reported [9,10], suggesting that other alterations in the active center are responsible for the lack of PARylation activity or that substrate specific effects restrict these enzymes to MARylation (Table 1).

**Table 1:** MARylation in signaling and disease

Protein	Enzymatic activity	Cellular process/ pathway	Disease-related function
ARTD3	MAR	DNA repair [11]	Not reported
ARTD4 (PARP4, vPARP)	MAR	Vault particles [12]	Multidrug resistance? [13,14,15,16] Innate immunity? [13]
ARTD7 (PARP15, BAL3)	MAR	Component of stress granules [17]	Not reported
ARTD8 (PARP14, BAL2, CoaSt6)	MAR	IL-4/STAT6 signaling [18,19,20] JNK signaling [21] T_H_ cell differentiation [22,23,24] Component of stress granules [17]	Promotes cell survival in B cells/multiple myeloma and lymphomagenesis [21,25,26] Allergic airway disease [22]
ARTD9 (PARP9)	inactive	IFNγ/STAT1 signaling [27,28,29]	Risk factor in DLBCL [27,28] Cell survival and chemoresistance [27,28,29]
ARTD10 (PARP10)	MAR	NF-κB signaling [30] Translation [31] Autophagy? [32] GSK3β signaling? [33]	Regulation of apoptosis [34] Inflammation? [30] IFN-stimulated gene, inhibition of viral replication [31]
ARTD12 (PARP12)	MAR	Translation [31] NF-κB signaling? [35] Autophagy? [35] Component of stress granules [17]	IFN-stimulated gene, inhibition of viral replication [31]
ARTD13 (PARP13, ZAP, ZC3HAV1)	inactive	mRNA regulation and miRNA silencing [36] Component of stress granules [17]	Viral defense [36]
ARTD14 (PARP7, TiPARP)	MAR	AHR signaling [37,38,39,40] Translation [31]	TCCD-induced hepatotoxicity [40] Inhibition of viral replication [31,41]
ARTD15 (PARP16)	MAR	Unfolded protein response [42]	Not reported
ARTD16 (PARP8)	MAR	Unclear	Not reported
ARTD17 (PARP6)	MAR	Regulates cell cycle progression [43]	Inhibits cell proliferation, survival benefit in colorectal cancer [43]
SIRT4	MAR	Glutamine metabolism [44,45]	Tumor-suppressive [45,46]
SIRT6	MAR	DNA repair [47,48,49] Retrotransposon silencing [50]	Tumor suppressive [50,51,52,53,54,55,56,57] and oncogenic functions [58,59]
MACROD1 (LRP16)	Hydrolase	ERα signaling [60,61] AR signaling [62]	Promotes cell proliferation [60,62] Metastasis, invasion and survival in gastric and colorectal cancer [63,64].
MACROD2 (C20ORF133)	Hydrolase	Unclear	Tamoxifen-resistance in breast cancer cell lines [65]
TARG1 (C6ORF130, OARD1)	Hydrolase	DNA repair? [66]	Neurodegeneration [66]

Besides ARTDs intracellular MARylation has also been ascribed to certain members of the Sirtuin family, namely SIRT4 and SIRT6 [44,67]. The mammalian Sirtuin family encompasses seven members, which have been originally identified to catalyze lysine deacetylation by the use of NAD^+^ as a co-factor thereby producing *O*-acetyl-ADP-ribose and nicotinamide [68,69]. A first indication that Sirtuins can function as mono-ARTs stems from the analysis of *Saccharomyces cerevisiae* Sir2, human SIRT2, and bacterial Sirtuins, which were shown to transfer ADP-ribose from NAD^+^ to substrates in biochemical assays [70,71]. More recently, SIRT4 and SIRT6 were described as mono-ARTs, thereby catalyzing MARylation [44,67]. 

So far, the evidence that MARylation catalyzed by mono-ARTDs and Sirtuins occurs in cells is limited due to the general difficulties to detect MARylation. While *in vitro* the use of radioactively labeled or chemically modified NAD^+^ can be used, the analysis in cells is more complex. Several antibodies are generally used to detect PAR chains synthesized in cells, but presently no comparable tools are available for MARylation. Moreover so far no consensus sequences could be defined, unlike e.g. with phosphorylation consensus sites identified for kinases, which has greatly facilitated their analysis. Thus the analysis of intracellular MARylation, *i.e.* the identification of substrates and modified sites, relies mainly on mass spectrometry (MS) approaches and on selective reader domains. The last years have shown considerable progress in the development of MS protocols that address the complexity of attachment sites with multiple amino acids described as potential sites of modification. Of note is also that ADP-ribose itself is challenging due to its behavior in different MS protocols and the potential to produce a number of different fragments making the analysis tedious. Moreover, the lack of efficient methods to analyze MARylation also poses a burden on the verification of MARylated substrates identified in various screens. It is important for the reader to realize these obstacles when discussing the functionality of intracellular MARylation. We refer to excellent recent reviews that discuss the detection and analysis of MARylation in detail [72,73,74]. 

The structural analysis of the enzymes of the ARTD sub-family revealed a multitude of other functional domains, likely reflecting the involvement of these enzymes in a variety of cellular processes [7,75]. In contrast the Sirtuins appear to be less complex. Besides the catalytic domain, both the N- and the C-terminal regions of SIRT4 and SIRT6 lack recognizable domains, suggesting that these enzymes use targeting subunits for selective activities [76]. Functions of polymer forming ARTDs and PARylation, with a variety of protein domains recognizing this PTM and enzymes capable of PAR degradation, are relatively well understood and range from the regulation of signaling and metabolism to the control of chromatin-related processes including transcription and DNA repair [3,77]. In contrast, the existence of ARTD enzymes being restricted to MARylation has only become apparent in the last decade, with ARTD10 being the founding member of this group [6]. Similarly, the identification of MARylation by Sirtuins is rather recent, as described above. In comparison to PARylation, relatively little is known about functions of MARylation so far and the lack of suitable tools for the detection of mono-ADP-ribosylated proteins, as detailed above, is impeding the analysis of intracellular MARylation and the identification of substrates. However, an increasing number of studies provides evidence that also MARylation serves as reversible PTM that can be read by specific protein domains (readers) and removed by MAR-specific hydrolases (erasers). Up-to-date, roles in the regulation of cell proliferation, apoptosis, signaling, metabolism, transcription and DNA repair have been described [1]. The so far identified reader and eraser proteins for intracellular MARylation share a common structural fold, the macrodomain. Indeed a role for macrodomains in ADP-ribose biology is becoming very well established. Some bind to free ADP-ribose or -derivatives, others exert catalytic activity towards ADP-ribosylated proteins or ADP-ribose derivatives. In addition some macrodomains show specificity for either MAR or PAR [78]. The macrodomains 2 and 3 of ARTD8, which are devoid of catalytic activity, have been identified recently as binding modules specific for at least some MARylated substrates [79]. So far, at least three enzymes are known to exist that are able to hydrolyze the bond between ADP-ribose and acidic acceptor amino acids, namely the two related MACROD1 and MACROD2, as well as C6ORF130/TARG1 [66,80,81]. Whether these hydrolases are active against all different MARylations, including those catalyzed by Sirtuins, has not been resolved. In the following we focus on the ARTD and Sirtuin family members with mono-ART activity and on the enzymes that can remove mono-ADP-ribose from substrate proteins. We review the roles of intracellular MARylation for the regulation of signaling pathways and discuss the relevance of this reversible PTM for disease. 

## 2. Regulation of Signaling Pathways and Gene Expression by MARylation 

### 2.1. ARTD8 Regulates IL4/STAT6 Signaling

**Figure 1:**
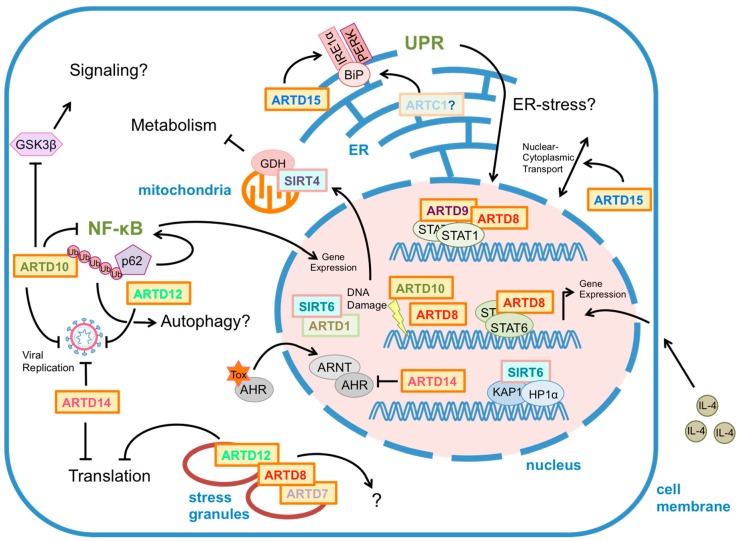
Functions of mono-ADP-ribosylating ARTD (ADP-ribosyltransferase diphtheria toxin-like) enzymes and of mono-ADP-ribosylating Sirtuins in signaling. Nuclear functions have been reported for ARTD8, ARTD9, ARTD10, ARTD14 and for SIRT6. ARTD8 has been described to regulate Signal Transducer and Activator of Transcription (STAT) 6-dependent gene expression, whereas ARTD8 and ARTD9 influence STAT1-dependent gene expression. Besides regulating STAT transcription factors, ARTD8 as well as ARTD10 and SIRT6 have been linked to DNA damage repair. ARTD14 interferes with AHR/ARNT (aryl hydrocarbon receptor/AHR nuclear translocator) driven gene expression. Moreover, SIRT6 regulates retrotransposon silencing. ARTD15 is localized to either the nuclear envelope where it influences nuclear-cytoplasmic shuttling or to the endoplasmic reticulum (ER) where it controls the unfolded protein response by modification of IRE1α (inositol requiring enzyme 1) and PERK (protein kinase RNA-like ER kinase). ARTD7, ARTD8 and ARTD12 are associated with stress granule formation. In addition, ARTD12 localizes to the autophagy receptor p62 and might be a player in the regulation of the NF-κB signaling pathway. Similarly, ARTD10 co-localizes with p62 in an ubiquitin-dependent manner and represses NF-κB signaling. ARTD10, ARTD12 and ARTD14 have been suggested to inhibit translation as well as viral replication. SIRT4 is localized to mitochondria and regulates glutamate dehydrogenase (GDH) activity and glutamine metabolism. For more details see the text.

ARTD8 (PARP14/BAL2/CoaSt6) was initially described as a member of the family of B-aggressive-lymphoma (BAL) proteins [82]. Then it was identified in a yeast two-hybrid screen as an interaction partner of Signal Transducer and Activator of Transcription (STAT) 6, co-activating IL-4-induced STAT6-dependent transcription. In this report, it was therefore termed CoaSt6 (collaborator of STAT6) (Figure 1) [18]. The IL-4/STAT6 signaling pathway is an important immune regulatory pathway affecting multiple cell types. STAT6-induced transcriptional programs mediate for example T_H_2 differentiation and allergic inflammatory T_H_2 effector responses in T cells as well as antibody class switching to the IgE isotype in B cells [83]. Upon binding of the cytokines IL-4 and IL-13 to receptors containing the IL-4Rα-subunit, STAT6 is subjected to JAK-mediated phosphorylation, resulting in homodimerization of phosphorylated STAT6 and its translocation into the nucleus, where it activates the transcription of target genes [83]. ARTD8 modulates this pathway depending on its macro- and C-terminal catalytic domains [18]. Later it was shown that ADP-ribosylation activity seems to be important for its co-activator function. A catalytically inactive mutant does not activate transcription from a STAT6 reporter plasmid and inhibition of ARTD activity by 3-aminobenzamide, an unspecific ARTD inhibitor, blocks IL-4 induced transcription [19]. According to the current model, ARTD8 acts as a “transcriptional switch” for STAT6-dependent transcription. In the unstimulated state, ARTD8 is associated with promoters of STAT6 responsive genes, interacting with the histone deacetylases HDAC2 and HDAC3, thereby repressing transcription. How precisely ARTD8 is recruited to such promoters has not been fully resolved. Stimulation of cells with IL-4 activates ARTD8 catalytic activity by an unknown mechanism, resulting in MARylation of HDAC2 and HDAC3. ARTD8 and HDACs are thereupon released from the promoter, allowing the STAT6 dimer to bind and activate transcription [20]. Thus these findings suggest that ARTD8 is a subunit of a transcriptional switch that controls HDAC activity and thus potentially histone acetylation in a promoter specific manner. Further studies will have to address the precise mechanism that is controlled by ARTD8 and in which cellular processes this switch is operational.

### 2.2. ARTD10 Modulates GSK3β Kinase Activity and NF-κB Signaling

ARTD10 has been identified in a screen for interaction partners of the oncoprotein c-MYC [84] and is the founding member of the group of mono-ARTDs [6]. Recent work revealed that ARTD10 plays important roles in the regulation of several cellular processes, including the control of cell proliferation, NF-κB signaling and DNA repair [85]. ARTD10 possesses a functional C-terminal catalytic domain, an N-terminally located RNA-recognition motif (RRM), glycine-rich and glutamate-rich regions, a nuclear export sequence (NES) and a region mediating nuclear uptake responsible for nuclear-cytoplasmic shuttling, and two ubiquitin-interaction motifs (UIMs) [84]. A protein array-based screen for ARTD10 substrates revealed that ARTD10 might target a variety of kinases as well as receptors and growth factors [33], which could account for the above-described biological effects. Several of these kinases have been validated in independent experiments, one of which is GSK3β, a serine/threonine-kinase regulating many cellular processes [86,87]. MARylation of GSK3β by ARTD10 *in vitro* and overexpression of ARTD10 in cells reduces GSK3βs kinase activity. Conversely, increased kinase activity can be observed after *ARTD10* knockdown and after overexpression of the MAR-specific hydrolase MACROD2, which was shown to remove MARylation from GSK3β *in vitro* [33,81]. Altogether, this provides first evidence that MARylation does indeed take place in cells and is able to regulate enzymatic activity. Whether ARTD10-dependent MARylation affects GSK3β-related signaling pathways, as for example WNT signaling, still needs to be investigated in future studies. Furthermore, the enrichment of ARTD10 (and ARTD8) substrates for kinases in the *in vitro* screen suggests that crosstalk exists between MARylation and other PTMs, including phosphorylation. ARTD10 itself is phosphorylated by multiple kinases, suggesting that it is controlled by several signaling pathways (our unpublished findings). 

With its two ubiquitin-interaction motifs, a unique feature of ARTD10 among the ARTDs, ARTD10 interacts with K63-linked poly-ubiquitin chains [30]. K63-linked poly-ubiquitin plays important roles for example in the NF-κB signaling pathway [88]. NF-κB proteins are dimeric transcription factors that regulate the expression of genes in response to stimulation of cells with pro-inflammatory cytokines including IL-1β and TNFα as well as pathogen-associated molecular patterns (PAMPs) such as lipopolysaccharide (LPS). In the unstimulated state, IκB sequesters NF-κB proteins in the cytosol. Upon activation of the signaling pathway, the IKK complex consisting of NEMO, IKKα and IKKβ is activated and phosphorylates IκB. IκB is then targeted for proteasome-mediated degradation, allowing for the release of the NF-κB p65/p50 dimer to translocate into the nucleus and activate the transcription of target genes [89]. ARTD10 has been shown to interfere with NF-κB signaling (Figure 1). In HeLa cells, the activation of the NF-κB target genes encoding for IκBα and IL-8 after stimulation with IL-1β is reduced upon overexpression of ARTD10, whereas ARTD10-GW/ΔUIM, a mutant that is catalytically inactive and fails to interact with K63-linked poly-ubiquitin, has no effect. Consistently, *ARTD10* knockdown in U2OS cells enhances the expression of these two NF-κB target genes. This is in agreement with ARTD10 acting as a repressor of NF-κB signaling dependent on its catalytic activity and on its ability to interact with K63-linked poly-ubiquitin. Intervention by ARTD10 is thought to take place at the level of NEMO poly-ubiquitination. K63-poly-ubiquitination of NEMO is essential for signal propagation and IKK-complex activation [90]. ARTD10 is probably recruited to K63-linked poly-ubiquitin chains via its two UIMs, promoting close contact with and subsequently MARylation of NEMO, thereby preventing its poly-ubiquitination and further downstream signaling [30]. 

### 2.3. ARTD14 Regulates AHR Signaling

ARTD14 (PARP7, TiPARP) has been identified in a screen for TCCD (2,3,7,8-Tetrachlorodibenzo-*p*-dioxin)-inducible genes [37]. TCCD is an environmental pollutant arising from industrial processes that elicits toxic responses in animals and humans. TCCD causes these effects by binding to the cytoplasmic aryl hydrocarbon receptor (AHR). Once activated by ligand binding, AHR translocates into the nucleus where it dimerizes with its binding partner ARNT (AHR nuclear translocator) and acts as a transcription factor. Target genes include cytochrome P450 encoding genes but also ARTD14 is induced upon TCCD treatment [37,38]. Further research has shown that ARTD14 is itself regulating AHR signaling in a negative feedback loop by acting as a transcriptional repressor (Figure 1) [39]. AHR can also serve as a substrate for ARTD14 [40]. However, whether ARTD14 regulates AHR activity or stability by MARylating waits to be investigated. It might promote proteasome-mediated degradation of AHR, be tethered to AHR response elements by interacting with AHR and thus interfere with a chromatin regulated process, or bind to the promoter directly mediated by its zinc-finger domain. The latter is suggested by the finding that its transcriptional co-repressor function depends on the zinc-finger domain and on the catalytic domain [39]. Interestingly, overexpressed MACROD1, but not MACROD2, is able to reverse the repressive effect of ARTD14 in a reporter gene assay and interacts with AHR [40]. Of note is that MACROD1 is mainly mitochondrial but the overexpressed protein is also detectable in the nucleus. Whether this is also true for the endogenous protein, e.g. in response to specific signals, has not been resolved. This suggests that MARylation by ARTD14 serves as an important PTM in the pathway controlling the response to environmental toxins such as dioxin and that MACROD1 might antagonize the repressive effect by removing MAR, either from ARTD14, AHR or other so far unidentified substrates. 

### 2.4. MARylation in the Unfolded Protein Response

Upon accumulation of unfolded proteins in the endoplasmic reticulum (ER) lumen, a process that elicits ER stress, the unfolded protein response (UPR) is activated [91,92]. Key players of the UPR sensing the overload of unfolded polypeptides are the two kinases IRE1α (inositol requiring enzyme 1) and PERK (protein kinase RNA-like ER kinase) as well as the transcription factor ATF6 (activating transcription factor 6) [91,92]. Luminal portions of IRE1α and PERK are associated with the chaperone BiP (binding immunoglobulin protein, also GRP78) [93]. Along with binding to increasing amounts of unfolded proteins during ER stress, BiP dissociates from IRE1α and PERK. The activation of these kinases during the UPR results in several different cellular consequences, including inhibition of translation to reduce the amount of incoming unfolded proteins as well as increased expression of proteins that help to promote the folding capacity of the ER [91,92,94]. ARTD15 (PARP16) was characterized as a mono-ADP-ribosyltransferase with a C-terminal transmembrane domain being localized to the ER membrane and the nuclear envelope (Figure 1) [42,95]. Accordingly, functions for ARTD15 have been described in nuclear-cytoplasmic transport as well as in the UPR [42,95]. *ARTD15* knockdown results in increased sensitivity to ER stress. ARTD15 is associated with IRE1α and PERK and activated under ER stress conditions resulting in MARylation of IRE1α and PERK in their cytosolic portions. Modification of both kinases with mono-ADP-ribose is increasing their kinase activity and enhancing downstream effects. Furthermore, in the ER lumen, the chaperone BiP remains bound to IRE1α and PERK when ARTD15 is depleted, arguing for a role of the luminal C-terminus of ARTD15 in BiP release [42]. An alternative model worth considering and testing is that the MARylation of these transmembranous kinases in the cytosolic portion induces a structural change in the luminal domain, which mediates interaction with BiP, resulting in reduced affinity. BiPs chaperone activity seems to be itself regulated by arginine-specific ADP-ribosylation. Modification of BiP at specific arginine residues was proposed to prevent interaction with unfolded proteins. The luminal BiP pool could thus be rapidly regulated by reversible ADP-ribosylation and serve as a buffer to quickly respond to changing levels of unfolded proteins in the ER [96]. Because ARTC enzymes passage through the ER en route to the outer leaflet of the plasma membrane and the extracellular space and because these enzymes modify arginines [4], it is possible that BiP proteins are modified by this sub‑family of mono-ARTs. Indeed, a recent study demonstrates ARTC1 to be mainly localized to the ER. During ER stress induction with dithiothreitol or thapsigargin, which results in a reduced protein flux into the ER, ARTC1 is transiently up-regulated. This promotes ADP-ribosylation and therefore inactivation of BiP [97]. The postulated rapid removal of the MARylation of BiP when the protein load is increasing again suggests that also a mono-ADP-ribosylhydrolase is present in the ER that will be interesting to identify. Together these studies define the ER as a subcellular site that involves MARylation in protein quality control. 

### 2.5. MARylation in Response to Genotoxic Stress

The role of PARylation in response to DNA damage is well studied. Mainly ARTD1 but also ARTD2 are sensing damaged DNA and reacting to the damage by auto-PARylation and PARylation of surrounding chromatin-associated proteins, in particular core histones [3]. PARylation leads to an opening of the chromatin and serves as a scaffold for the recruitment of repair factors, enabling efficient DNA repair. Knowledge about the function of ADP-ribosylation in DNA repair is so far mainly restricted to the polymer-forming ARTD family members. However, several recent reports provide insights into a role of MARylation in response to genotoxic stress. Knockdown of *ARTD10* in HeLa cells results in enhanced sensitivity to DNA damage induced by mitomycin C, hydroxyurea (HU) or UV light treatment. Increased γ-H2AX, phospho-RPA and RAD51 foci formation has been observed under *ARTD10* knockdown conditions [98]. These are markers to indicate DNA damage and because of the prolonged foci formation reduced DNA repair is assumed in conditions with reduced ARTD10 expression. As a component of the replication machinery, PCNA acts as a master regulator of replication and coordinates DNA repair and resumption of DNA replication at stalled replication forks [99]. ARTD10 was shown to interact with PCNA mediated by a region in ARTD10 termed PIP-box. The interaction with PCNA is further strengthened by interaction of ARTD10’s UIMs with ubiquitinated PCNA [98]. ARTD10 seems to be important for the restart of the DNA replication machinery after replication fork stalling. ARTD10 is important for maintaining PCNA ubiquitination levels by an as yet unknown mechanism, favoring the recruitment of error-prone translesion synthesis polymerases to stalled replication forks. Catalytic activity seems to be important for this process although relevant substrates have not been identified so far [98]. In addition to ARTD10, ARTD8 interacts with PCNA in cells, preferentially when these are in the S-phase of the cell cycle [100]. Similar to ARTD10, ARTD8 depletion enhances the sensitivity of cells to replication stress upon HU or UV treatment and accordingly more cells exhibit γ-H2AX foci formation in response to HU/UV treatment. ARTD8 is as well important for the restart of replication after replication fork stalling. In contrast to ARTD10, ARTD8 regulates DNA strand break repair via homologous recombination (HR). Interaction of one of the MAR-specific reader domains of ARTD8, macro2, with RAD51 was demonstrated. It is argued that RAD51 acts as an ARTD8 substrate during HR and thus the interaction with macro2 might be part of a feedback loop. How relevant these interactions are for proper HR repair remains to be investigated [100].

Besides ARTD8 and ARTD10, SIRT6 has also been linked to genotoxic stress [47]. Although most of its molecular functions, e.g. in the regulation of stress-responsive and metabolism-related genes or its roles in base excision repair and double strand break (DSB) repair, have been ascribed to its deacetylase activity so far [101,102,103], recent research has linked the MARylation activity of SIRT6 to DNA repair [47,48]. SIRT6 maintains genome integrity by regulating DNA repair underlined by the fact that cells lacking SIRT6 are hampered to repair DSBs and mice devoid of SIRT6 exhibit impaired base excision repair [49,104]. Under oxidative stress SIRT6 is recruited to sites of DNA damage where it stimulates non-homologous end joining as well as HR. SIRT6 mutants allowing the distinction of its deacetylase activity from its MARylation activity identified the latter to be important for its role in DSB repair. SIRT6 was shown to interact with and MARylate ARTD1, which in turn triggers the catalytic activity of ARTD1 and promotes DSB repair. Thus it appears that SIRT6 contributes to DSB repair indirectly through activating ARTD1 by MARylation [47]. In line with this, SIRT6 is also able to rescue the age-related decline in HR efficiency dependent on its ability to catalyze MARylation of ARTD1 [48].

Among the recently identified macrodomains capable of hydrolyzing MAR modifications, MACROD2 and C6ORF130/TARG1 have been shown to localize to sites of DNA damage when overexpressed [66,80]. TARG1 is important for DNA repair as cells depleted of TARG1 show increased sensitivity to DNA damaging agents. This suggests that these two enzymes are involved in regulating ADP-ribosylation in response to DNA damage. A possible scenario might be that to remove PARylation when ARTD1 was activated, first poly-ADP-ribosylglycohydrolase (PARG) and/or ADP-ribosylhydrolase 3 (ARH3) remove the polymers until the most proximal ADP-ribose unit. Because both enzymes cannot remove the ADP-ribose bound to the protein, the final step in completely removing PARylation requires MACROD2 and/or TARG1 or potentially other as yet unidentified enzymes. Furthermore MACROD2 and TARG1 are directly implicated in antagonizing the activities of ARTD8 and ARTD10, which are involved in DNA repair as discussed above. However, it should be noted that TARG1 may have a weak ability to revert PARylation directly by removing PAR chains [66]. These enzymes therefore seem to play a role for the (complete) turnover of ADP-ribosylation at DNA damage sites. They may also prevent over-activation of ARTD1 by counteracting initial ARTD1 auto-MARylation. 

Genomic instability and the acquisition of mutations are characteristic steps in tumorigenesis and can be caused by defects in proteins or pathways important for the detection or repair of damaged DNA. In light of the above-described recent findings concerning the functions of MARylation in DNA repair, writers and/or erasers of MARylation might as well contribute to cancer development when deregulated or serve as therapeutic targets in the treatment of cancer, as it is already the case for their polymer-forming relatives [105].

## 3. MARylation in Disease

### 3.1. Cancer and Metabolism

NAD^+^ is an essential component of different cellular processes, including glycolysis and the transfer of electrons from NADH to the mitochondrial respiratory chain. In tumor cells, increased glycolysis is observed owing the highly increased demand of biomass [106,107]. To provide sufficient levels of NAD^+^, it is regenerated from NADH by a process catalyzed by lactate dehydrogenase, reducing pyruvate to lactate [108]. Thus NAD^+^ is closely associated with metabolic pathways not only in normal cells but particularly also in tumor cells. ARTDs and Sirtuins consume NAD^+^ in the process of ADP-ribosylating substrates, requiring a salvage pathway to recycle nicotinamide as well as a de novo synthesis pathway [109]. With these findings in mind it is not unexpected that enzymes that consume NAD^+^ are potentially involved in controlling metabolism and tumorigenic cell proliferation. 

Several reports indicate that MARylation is important for the regulation of cell proliferation [105,110]. Among those members of the ARTD family that exert MARylation activity, especially ARTD8 and ARTD10 have been demonstrated to modulate cell survival or apoptosis [25,26,34,84,105,110]. Moreover ARTD9, a catalytically inactive member of ARTDs, has been shown to regulate signaling as detailed below, suggesting that these ARTD members could contribute to cancer formation when deregulated. 

ARTD9 (PARP9/BAL1) was described as a risk factor for diffuse large B cell lymphoma (DLBCL) and therefore initially referred to as BAL1 (B-aggressive lymphoma 1) [27]. Although ARTD9 has been demonstrated to be catalytically inactive based on *in vitro* automodification studies [82], which is supported by structural considerations [6], it acts as an oncogenic factor in DLBCL [28] and possibly in metastatic prostate cancer cell lines [29]. In DLBCL, ARTD9 modulates interferon (IFN) γ-STAT1 signaling in a way that induces a transcriptional switch resulting in the repression of tumor suppressor genes such as *IRF1* and activation of proto-oncogenes. ARTD9 has two N-terminal ADP-ribose-binding macrodomains [27]. These macrodomains are responsible for interaction of ARTD9 with STAT1 and for its repressive effect on an *IRF1* promoter-based luciferase reporter assay, suggesting that ADP-ribosylation of STAT1 mediates the interaction with ARTD9 [28]. In metastatic prostate cancer cell lines, survival and chemoresistance in response to IFNγ are mediated by ARTD9 with its interaction partner DTX3L/BBAP in a STAT1-dependent way [29]. In addition, ARTD8 acts as a survival factor in these cell lines. ARTD8 was initially described as a BAL family member because of the structural similarities, *i.e.* the presence of both an ADP-ribosyltransferase domain and macrodomains, to ARTD9/BAL1 [82]. ARTD8 interacts with both DTX3L and ARTD9 but does not regulate the before mentioned IFNγ-STAT1-IRF1 axis. Thus whether ARTD8 is the enzyme that MARylates STAT1, which subsequently is read by ARTD9, remains to be determined.

Furthermore, ARTD8 acts as a pro-survival factor in B cells. As a co-regulator of IL-4/STAT6-signaling, ARTD8 mediates IL-4-induced inhibition of apoptosis in B cells by regulating the expression of survival factors, repressing caspase-dependent apoptotic processes and regulating glycolytic rates [25,26]. PJ-34 treatment protects B cells from apoptosis [25]. Although PJ-34 is an unspecific ARTD inhibitor, this suggest that ADP-ribosylation activity, possibly that of ARTD8, is involved in this process. Additionally, ARTD8 deficiency attenuates MYC-induced B cell lymphoma development in mice [26]. ARTD8 may also impact JNK signaling in multiple myeloma. It has been observed that constitutively active JNK2 suppresses JNK1-mediated apoptosis in these tumors [21]. This effect is mediated by ARTD8, whose expression depends on JNK2, and interacts with JNK1 resulting in inhibition of its kinase activity. The interaction with JNK1 is restricted to ARTD8’s C-terminal region comprising the WWE and the catalytic domain [21]. Whether MARylation of, for example, JNK1 by ARTD8’s catalytic domain is important for JNK1 inhibition has not been investigated in detail. But as JNK1 activation and accordingly apoptosis of multiple myeloma cells occurs after treatment with PJ-34, catalytic activity of ARTD8 might be important in this process [21]. Altogether, this indicates that ARTD8 might be relevant as a therapeutic target in B cell malignancies and prostate cancer. 

In contrast to ARTD8, a first characterization of ARTD10 pointed to a growth-inhibitory role. ARTD10 was identified as an interaction partner of the oncoprotein c-MYC. In primary rat embryonic fibroblasts, ARTD10 inhibits MYC/Ha-RAS- and E1A/Ha-RAS-induced transformation independent of catalytic activity [84]. Unlike in these primary cells, in HeLa cells ARTD10 but not the catalytically inactive ARTD10-G888W mutant inhibits cell proliferation [6,34]. HeLa cells stably expressing ARTD10 are driven into apoptosis, probably mediated by MARylation of (so far not identified) ARTD10 substrate(s). During apoptosis induction, ARTD10 is exposed to caspase-6-mediated cleavage at D406 inactivating its pro-apoptotic properties [34]. As a non-cleavable mutant of ARTD10 induces efficiently apoptosis and the cleavage products alone do not, it was suggested that the N-terminally located RRMs and the C-terminally located catalytic domain cooperate during apoptosis induction. How ARTD10 induces apoptosis mechanistically still remains to be clarified. Interestingly, knockdown of *ARTD10* in HeLa cells results in growth inhibition as well [111], suggesting that a tight regulation of ARTD10 levels in cells is indispensable for normal cell growth. Interaction with c-MYC and its above-mentioned repressive role in NF-κB signaling might by relevant to control cell proliferation and apoptosis. The inflammatory NF-κB pathway is activated in many types of hematological malignancies and solid cancers. As a consequence, pro-proliferative and anti-apoptotic NF-κB target genes are induced, promoting tumor development [89,112]. To what extend ARTD10 contributes to the pro-tumorigenic activities of NF-κB is a matter of current research. 

Besides ARTD8 and ARTD10, ARTD17 seems to be a regulator of cell proliferation. Overexpression of ARTD17 in HeLa cells inhibits cell growth dependent on the integrity of the catalytic domain and patients with ARTD17-positive colorectal cancer show higher survival rates compared to ARTD17-negative colorectal cancer patients [43].

ARTD4 has been identified as a component of vault particles [12]. Vaults are barrel-shaped ribonucleoprotein particles consisting of untranslated vault RNA molecules and the three proteins MVP (major vault protein), ARTD4 (vPARP, PARP4) and TEP1 (telomerase associated protein 1). The functions of vault particles are still not fully clear but roles in cellular transport, signaling, immunity and multidrug resistance have been discussed [13,14]. The exact role of ARTD4 in vault particles is unknown, but due to its up-regulation in some drug resistant cancer cell lines [15,16] one might speculate about an involvement of ARTD4 in the development of drug resistance in cancer. In addition to vault particles, ARTD4 localizes to the nucleus and the mitotic spindle [12], so vault-independent functions are likely. Furthermore, ARTD4-deficient mice are prone to carcinogen-induced colon tumor formation [113]. 

In addition to these members of the ARTD family, recent studies revealed that SIRT4 possesses tumor suppressor function [44,45,46], whereas SIRT6 has been reported to have both tumor suppressive and promoting functions [102]. SIRT4 is located to mitochondria and displays ADP-ribosyltransferase activity rather than deacetylase activity [44]. SIRT4 has been demonstrated to interact with and MARylate glutamate dehydrogenase (GDH), a metabolic enzyme catalyzing the conversion of glutamate to α-ketoglutarate, an intermediate of the TCA cycle (Figure 1) [44]. Thus, by negatively modulating the activity of GDH SIRT4 exerts a role in glutamine metabolism. Recently glutamine metabolism has been linked to genotoxic stress. DNA damage promotes upregulation of SIRT4 expression, which subsequently results in downregulation of mitochondrial glutamine turnover [45]. Because glutamine metabolism is important for proliferation, particularly of tumor cells, and required for cell cycle transition from G_1_ to S phase, the cell cycle is arrested in consequence of reduced glutamine conversion [107]. This suggests that SIRT4 contributes to a metabolic checkpoint by regulation of GDH in response to genotoxic stress [45]. Besides this SIRT4 is also critical for genome integrity e.g. by facilitating clearance of γ-H2AX foci which arise at sites of DNA damage [45]. In line with this, SIRT4 is able to suppress tumor formation, whereas the loss of SIRT4 enables tumor formation at least in part by elevated glutamine metabolism, loss of the metabolic checkpoint and as a consequence genomic instability accompanied by enhanced proliferation. Indeed this has been observed in several tumor entities, including gastric adenocarcinomas, colorectal cancer and bladder cancer [45,114,115,116].

SIRT6 has been reported to have tumor suppressive functions. Its expression is reduced in several types of cancers [51,52,53,54,55]. Accordingly, overexpression of SIRT6 in different cancer cell lines reduces cell proliferation and induces apoptosis [51,52,53,56] Although some of these effects seem to be mediated by its deacetylase activity [55,57], SIRT6’s MARylation activity might as well be responsible for apoptosis induction [56]. Recently, MARylation activity of SIRT6 has been demonstrated to be important for transcriptional silencing of transposable elements. SIRT6 is associated with the 5’-UTR of L1 retrotransposons. SIRT6 regulates the recruitment of KAP1 and HP1α, factors associated with heterochromatin [50]. MARylation of KAP1 by SIRT6 promotes interaction between KAP1 and HP1α, thereby promoting the recruitment of HP1α to the L1 5’-UTR. This results in packaging of these retrotransposons into repressive heterochromatin [50]. During aging and in response to genotoxic stress, SIRT6 association with L1 is reduced or even lost. This activates the L1 retrotransposons, which can ultimately contribute to genomic instability and age-related diseases such as cancer [50]. It should be noted, however, that some reports state a more oncogenic function for SIRT6 [58,59]. Thus SIRT6 might have distinct functions depending on the type of cancer.

Roles in the regulation of cell proliferation, cancer development and drug resistance have also been ascribed to the three so far identified MAR hydrolases. Several studies point to an association of MACROD1 (LRP16) with cancer. MACROD1 expression is induced by estrogen/estrogen receptor alpha (ERα) signaling in ERα-positive breast cancer cell lines [60,117]. Overexpression of the estrogen receptor and activated ERα signaling is observed in certain breast cancer subtypes but also in other tumors. Sustained activation of ERα signaling stimulates proliferation of mammary cells, eventually leading to tumor formation [118]. MACROD1 interacts with ERα and functions in a positive feedback-loop as a co-activator of ERα-dependent transcription, enhancing the expression of several ERα target genes, including *c-MYC* and *CCND1*, thereby provoking increased cell proliferation [60,61,117,119]. In analogy to its role in ERα signaling, MACROD1 interacts with and stimulates the transcriptional activity of the androgen receptor (AR) and in AR responsive prostate cancer cells, MACROD1 is needed for cell proliferation stimulated by testosterone [62]. Because the above described findings all rely on MACROD1’s macrodomain, which was recently shown to possess hydrolase activity specifically towards MARylation, one might speculate about a general involvement of MARylation in steroid hormone receptor signaling. Overexpression of MACROD1 might be associated with increased invasion, metastasis and shorter survival time in gastric and colorectal cancer [63,64]. Interestingly, a recent publication demonstrates that tamoxifen-resistance in breast tumors can be mediated by the related MACROD2 [65]. The authors observed amplification and overexpression of *MACROD2* in tamoxifen-resistant breast cancer cell lines and in human breast cancer samples. *MACROD2* knockdown sensitizes tamoxifen resistant cells to tamoxifen treatment and reduces tumor formation of tamoxifen resistant cells in a xenograft model, suggesting that MACROD2 could become an important molecular target in the treatment of tamoxifen resistant breast cancer.

Together the studies summarized above indicate involvement of enzymes controlling MARylation in cancer (Table 1). However, it is not clear whether any of the writers and erasers of MARylation function as oncoproteins or as tumor suppressors. Therefore we evaluated the mutational alterations associated with the different genes associated with MARylation, *i.e.* the mono-ARTs and -hydrolases, using COSMIC (Catalogue Of Somatic Mutations In Cancer; the analysis was performed on 03 July 2015) [120]. All the genes analyzed (ARTD3, 4, 7–17, SIRT4, SIRT6, MACROD1, MACROD2, C6ORF130) are mutated at low frequency throughout their entire coding regions of more than 21.000 tumors in the database. No accumulation of mutations in one particular tumor type is observed. Also there is no trend for specific protein regions being mutated such as the catalytic domains. This suggests that none of the analyzed genes is a hotspot for mutations in cancer. Also no translocations have been reported associated with these genes. Few cases with deletions or insertions are found in the COSMIC database but again no recurrent patterns are seen that would suggest these mutations being of patho-physiological importance.

Altered gene expression, with either a two-fold up- or down-regulation, is in most instances also rare. This goes with typically few copy number variant (CNV) gains or losses. However, a few findings are worth reporting as these might give indications for a contribution of a specific gene in certain tumors. For example *ARTD3* is overexpressed in more than 30% of tumors of the large intestine but is underexpressed in about 26% of ovarian cancer. *ARTD9*, which is a risk factor for DLBCL, is up-regulated in many tumors with frequencies of up to 10%. *ARTD10* is overexpressed in between 20 and 30% of liver, esophagus, and ovarian cancer, and between 10 and 20% in cancer of the adrenal gland, the cervix, the stomach, and in head and neck squamous cell carcinoma. *ARTD14* is overexpressed in more than 20% of esophageal cancer and in these tumors more than 12% show a CNV gain, suggesting that ARTD14 might be of functional relevance in esophageal cancer. The other genes are typically only affected in 10% or less cases of a given specific tumor. Down-regulation of these genes is observed very rarely and thus a role in tumor suppression is rather unlikely. Together, these findings suggest that the writers and readers of MARylation are affected in tumors but the available data does not support a strong oncogenic or tumor suppressor function associated with these genes and proteins at present. 

### 3.2. Innate Immunity and Inflammation

#### 3.2.1. Mono-ARTDs in the Defense against Viral Infections

The innate immune system represents the first line of defense against invading pathogens and operates during the first days of infection. It recognizes the nature of the invading pathogen through interactions of PAMPs and their respective cellular pattern recognition receptors and is able to activate adaptive immune responses [121]. A crucial component of innate immunity is cytokine-mediated signaling with the aim to inform and alarm neighboring (and immune) cells of the infection and induce defense mechanisms in an auto- and paracrine fashion. IFNs are cytokines produced upon viral infections with the aim to induce anti-viral responses via the induction of IFN-stimulated genes. Several mono-ARTDs are induced upon stimulation with type I IFNs and exert anti-viral effects. *ARTD12* expression is induced by Toll-like receptor stimulation through LPS or IFNβ [35]. During Venezuelan Equine Encephalitis Virus (VEEV) infection, *ARTD12* expression is up-regulated in a type I IFN-dependent manner [41]. The long isoform of ARTD12 as well as ARTD10 and ARTD14 inhibit replication of several RNA viruses like alphaviruses [31,41]. In addition, cellular translation is blocked after overexpression of these mono-ARTDs, which might account at least in part for an inhibition of viral replication. This is probably mediated by a concerted action of putative RNA binding motifs and catalytic domains as these three enzymes share a combination of such functional domains. As both ARTD10 and ARTD12 have been shown to localize to cytoplasmic structures containing the autophagic membrane adaptor protein p62, it has been speculated that both proteins regulate autophagic processes and might therefore affect pathogen clearance via autophagy [32,35]. This may be further affected by ARTD10 controlling NF-κB, which has also been implicated in the regulation of autophagy [30,122]. Moreover ARTD12 is able to induce expression of an NF-κB-responsive luciferase reporter plasmid and of endogenous NF-κB target genes [35]. As activation of NF-κB signaling is a key process in inflammation, MARylation by ARTD10 and ARTD12 might therefore modulate immune responses by targeting this pathway. 

A recent report demonstrated that several mono-ARTDs have evolved under positive selection [123]. This includes ARTD4, the three macrodomain-containing ARTDs, ARTD7, -8 and -9, and the catalytically inactive ARTD13 and indicates a direct involvement of these proteins in the defense against pathogens during recurrent conflicts of hosts with viral but also bacterial or eukaryotic pathogens. As mentioned above, ARTD4 is part of vault particles, which are linked to immunity and viral infection. Although precise functions of ARTD4 in these processes have not been described, it is hypothesized that this ARTD member is also involved in the anti-viral response [123]. ARTD13 (ZAP; zinc finger anti-viral protein) occurs in two isoforms. The short isoform lacks a C-terminal ART domain, whereas this domain is present but catalytically inactive in the long isoform [6]. ARTD13 inhibits the replication of certain retro-, alpha-, filo- and hepadnaviruses [124,125,126,127,128]. Through its four CCCH-type zinc finger domains it binds viral mRNAs [129,130,131] and recruits the cellular RNA degradation machinery [126,128,131,132], thereby preventing the translation of viral RNAs and viral replication. Thus, the anti-viral activity of ARTD13 is mainly accomplished by its N-terminal domain. However, the ARTD-like domain present in the long isoform might play a role in restricting the replication of alphaviruses [133]. 

Although a direct role of ADP-ribosylation by ARTD13 can be excluded because ADP-ribosylation activity is lacking, crosstalk between other members of the ARTD family and ARTD13 might contribute to its cellular functions. Lately, ARTD13 was demonstrated to localize to stress granules (SGs) along with ARTD5, -7, -8, -12 and PARG [17]. SGs are dynamic ribonucleoprotein aggregations of translationally stalled mRNAs, ribosomal subunits and different RNA binding proteins [134]. They form upon different forms of cellular stress including oxidative stress, heat shock or nutrient starvation and regulate the stability or translation of mRNAs [134]. Overexpression of the SG-associated ARTDs is sufficient to induced SG assembly and several SG components are modified with ADP-ribose, as for example Ago2. Its modification by ADP-ribosylation is enhanced in response to stress [17]. Furthermore, ARTD13 regulates miRNA silencing [17]. MiRNA-mediated repression of target mRNAs is decreased after ARTD13 overexpression, *PARG* knockdown and upon stress. All of these conditions result in increased modification of Ago2 with ADP-ribose but also of ARTD13 itself [17]. This is established by interactions between ARTD13 and ARTD5, -7 and -12, as well as between Ago2 and ARTD5 and -12, and involves in *trans* mono- and poly-ADP-ribosylation by ARTD5, -7 and -12 [17,135]. PARG reverses these effects [17]. A tight regulation of MARylation and PARylation involving mono- and poly-ARTDs as well as PARG therefore seems to be important for the assembly of SGs and the function of miRNP complexes. As sites of silenced mRNA, SGs have been discussed regarding their role in viral infections [136,137]. In addition to the different forms of cellular stress that trigger SG assembly, SGs can be induced during infection of cells with certain viruses. Contrarily, some viruses actively inhibit SG formation during infection, suggesting that SGs can play an active role in anti-viral defense [136,137]. Although many open questions concerning the interplay of SGs and invading viruses need to be clarified in the future, one might speculate about a function of SG-associated ARTDs in viral defense by promoting SG formation [136,137].

Altogether this provides initial evidence that intracellular mono-ARTDs and MARylation play an important role in the host’s immune defense against infections. Accordingly, viruses might have developed mechanisms to evade the host’s immune defense involving ADP-ribosylation. RNA viruses of the Toga- and Coronaviridae families (for example alphaviruses including VEEV, Chikungunya virus (CHIKV), Sindbis virus (SINV); rubella virus; hepatitis E virus (HEV) and SARS-Coronavirus (SARS-CoV)) encode one or more macrodomains as part of non-structural proteins that are capable of binding to ADP-ribose and PAR [138,139]. In some cases, these domains are able to hydrolyze ADP-ribose-1′′-phosphate [138,140]. This raises the possibility that viral macrodomains could act as hydrolases for ADP-ribose modifications. A role of the viral macrodomains in counteracting the defense of host cells is strengthened by the fact that mutations in viral macrodomains result in a reduced virulence in mice [141,142] and mutation of the SARS-CoV macrodomain confers increased sensitivity to IFN treatment [143]. To what extent these macrodomains interfere with ADP-ribosylation in the host cell needs to be investigated in future studies.

#### 3.2.2. Bacterial mono-ADP-ribosyltransferases 

As indicated above, SIRT4 and SIRT6 are two mammalian Sirtuins, which have been demonstrated to possess MARylation activity. It is interesting to note that also bacterial Sirtuins have been described as mono-ADP-ribosylating enzymes [70,144]. Recently substrate modification by the Sirtuins of *Staphylococcus aureus* and *Streptococcus pyogenes*, which are of a distinct Sirtuin subclass referred to as SirTMs, was demonstrated to be dependent on another PTM. Prior lipoylation of the substrate is necessary for efficient MARylation by these enzymes [144]. These *Sirtuin* genes are genetically linked to genes expressing macrodomain proteins, which are capable to revert SirTM-specific MARylation. The precise function of these mono-ADP-ribosyltransferase–hydrolase pairs remains to be determined. However, with the increasing accumulation of information that links MARylation to immune response and anti-viral activities, it is of interest to speculate that also bacterial pathogens use this PTM to counteract host defense. 

In addition to the rather recently discovered and still poorly explored bacterial Sirtuins, bacterial ARTs have long been known to affect cellular behavior by ADP-ribosylating specific host cell target proteins. Bacterial pathogens use toxins, including ARTs, to alter and kill host cells. A main purpose of these toxins is to disrupt immune cell functions and by this promote pathogen dispersal and proliferation, which is summarized in excellent reviews [145,146].

#### 3.3.3. ARTD8 Modulates T_H_-Cell Differentiation

The function of ARTD8 in STAT6-dependent transcriptional processes might be of physiological relevance for T and B cell responses *in vivo*. Deregulation of the T_H_2 response caused by disturbed IL-4/STAT6-signaling can contribute to the pathogenesis of inflammatory lung diseases such as asthma [83]. Reduced levels of T_H_2-specific cytokines IL-4, IL-5 and IL-13 were observed in *in vitro* differentiated T_H_2 cells isolated from ARTD8-deficient mice and after *ARTD8* knockdown [22]. A model was proposed, in which ARTD8 regulates binding of STAT6 to the GATA3 promoter, thereby modulating T helper cell differentiation. Furthermore, the induction of allergic airway disease in this ARTD8 knockout mouse model resulted in immune cell infiltration into the lung associated with reduced levels of T_H_2 cytokines and chemokines as well as reduced IgE levels, arguing for a pro-allergic role of ARTD8. Moreover general inhibition of ARTD enzymatic activity by PJ-34 reduced signs of the inflammatory T_H_2 response, although it has not been clarified if this effect is ascribed to ARTD8 inhibition only or if inhibition of other ARTD family members contributes to this phenotype. A possible role for ARTD8 in T_H_2 and IL-4-induced differentiation as well as in the IgE switch in B cells was also observed [23]. In addition, this group found that T_H_17 differentiation and accordingly production of IL-17 and -22 is impaired in ARTD8-deficient T cells as well depending on its catalytic activity, in turn affecting the induction of IgA responses in B cells [23,25]. If MARylation of HDAC2/3 and p100, transcriptional co-regulators of STAT6 (as described above), or MARylation of additional substrates plays a role in these processes, has not been investigated in these studies.

## 4. Conclusions

The last several years have provided a number of lines of evidence to support the hypothesis that MARylation is involved in regulating several different physiological processes that include signaling pathways, gene transcription, DNA repair, and stress control. This is most likely the tip of the iceberg and we expect to see more evidence for regulatory functions of MARylation and the enzymes that control this PTM, *i.e.* the mono-ADP-ribosyltransferases and -hydrolases. In addition to studying the enzymes, we will need to understand how MARylation affects the function of target proteins and how the information is transmitted to other cellular components. One aspect of this is to define the readers of MARylation. Some macrodomains can bind to MARylated proteins but it seems likely that additional domains exist that connect this PTM to other cellular processes. Together interesting times are ahead of us that will shed more light on the regulatory roles MARylation plays in controlling cell physiology and organismal functions potentially associated with disease.

## References

[1] Feijs K.L., Verheugd P., Luscher B. (2013). Expanding functions of intracellular resident mono-ADP-ribosylation in cell physiology. Febs J..

[2] Feldman J.L., Dittenhafer-Reed K.E., Denu J.M. (2012). Sirtuin catalysis and regulation. J. Biol. Chem..

[3] Gibson B.A., Kraus W.L. (2012). New insights into the molecular and cellular functions of poly(ADP-ribose) and PARPS. Nat. Rev. Mol. Cell. Biol..

[4] Glowacki G., Braren R., Firner K., Nissen M., Kuhl M., Reche P., Bazan F., Cetkovic-Cvrlje M., Leiter E., Haag F. (2002). The family of toxin-related ecto-ADP-ribosyltransferases in humans and the mouse. Protein Sci..

[5] Seman M., Adriouch S., Haag F., Koch-Nolte F. (2004). Ecto-ADP-ribosyltransferases (arts): Emerging actors in cell communication and signaling. Curr Med. Chem..

[6] Kleine H., Poreba E., Lesniewicz K., Hassa P.O., Hottiger M.O., Litchfield D.W., Shilton B.H., Luscher B. (2008). Substrate-assisted catalysis by parp10 limits its activity to mono-ADP-ribosylation. Mol. Cell.

[7] Hottiger M.O., Hassa P.O., Luscher B., Schuler H., Koch-Nolte F. (2010). Toward a unified nomenclature for mammalian ADP-ribosyltransferases. Trends Biochem. Sci..

[8] Karlberg T., Klepsch M., Thorsell A.G., Andersson C.D., Linusson A., Schuler H. (2015). Structural basis for lack of ADP-ribosyltransferase activity in poly(ADP-ribose) polymerase-13/zinc finger antiviral protein. J. Biol. Chem..

[9] Loseva O., Jemth A.S., Bryant H.E., Schuler H., Lehtio L., Karlberg T., Helleday T. (2010). Parp-3 is a mono-ADP-ribosylase that activates PARP-1 in the absence of DNA. J. Biol. Chem..

[10] Vyas S., Matic I., Uchima L., Rood J., Zaja R., Hay R.T., Ahel I., Chang P. (2014). Family-wide analysis of poly(ADP-ribose) polymerase activity. Nat. Commun..

[11] Beck C., Robert I., Reina-San-Martin B., Schreiber V., Dantzer F. (2014). Poly(ADP-ribose) polymerases in double-strand break repair: Focus on PARP1, PARP2 and PARP3. Exp. Cell. Res..

[12] Kickhoefer V.A., Siva A.C., Kedersha N.L., Inman E.M., Ruland C., Streuli M., Rome L.H. (1999). The 193-kd vault protein, vparp, is a novel poly(ADP-ribose) polymerase. J. Cell. Biol..

[13] Berger W., Steiner E., Grusch M., Elbling L., Micksche M. (2009). Vaults and the major vault protein: Novel roles in signal pathway regulation and immunity. Cell. Mol. Life Sci..

[14] Mossink M.H., van Zon A., Scheper R.J., Sonneveld P., Wiemer E.A. (2003). Vaults: A ribonucleoprotein particle involved in drug resistance?. Oncogene.

[15] Siva A.C., Raval-Fernandes S., Stephen A.G., LaFemina M.J., Scheper R.J., Kickhoefer V.A., Rome L.H. (2001). Up-regulation of vaults may be necessary but not sufficient for multidrug resistance. Int. J. Cancer.

[16] Schroeijers A.B., Siva A.C., Scheffer G.L., de Jong M.C., Bolick S.C., Dukers D.F., Slootstra J.W., Meloen R.H., Wiemer E., Kickhoefer V.A. (2000). The mr 193,000 vault protein is up-regulated in multidrug-resistant cancer cell lines. Cancer Res..

[17] Leung A.K., Vyas S., Rood J.E., Bhutkar A., Sharp P.A., Chang P. (2011). Poly(ADP-ribose) regulates stress responses and microrna activity in the cytoplasm. Mol. Cell.

[18] Goenka S., Boothby M. (2006). Selective potentiation of Stat-dependent gene expression by collaborator of Stat6 (CoaSt6), a transcriptional cofactor. Proc. Natl. Acad. Sci. USA.

[19] Goenka S., Cho S.H., Boothby M. (2007). Collaborator of stat6 (CoaSt6)-associated poly(ADP-ribose) polymerase activity modulates Stat6-dependent gene transcription. J. Biol. Chem..

[20] Mehrotra P., Riley J.P., Patel R., Li F., Voss L., Goenka S. (2011). Parp-14 functions as a transcriptional switch for Stat6-dependent gene activation. J. Biol. Chem..

[21] Barbarulo A., Iansante V., Chaidos A., Naresh K., Rahemtulla A., Franzoso G., Karadimitris A., Haskard D.O., Papa S., Bubici C. (2013). Poly(ADP-ribose) polymerase family member 14 (parp14) is a novel effector of the Jnk2-dependent pro-survival signal in multiple myeloma. Oncogene.

[22] Mehrotra P., Hollenbeck A., Riley J.P., Li F., Patel R.J., Akhtar N., Goenka S. (2013). Poly (ADP-ribose) polymerase 14 and its enzyme activity regulates T(H)2 differentiation and allergic airway disease. J. Allergy. Clin. Immunol..

[23] Cho S.H., Raybuck A., Wei M., Erickson J., Nam K.T., Cox R.G., Trochtenberg A., Thomas J.W., Williams J., Boothby M. (2013). B cell-intrinsic and -extrinsic regulation of antibody responses by parp14, an intracellular (ADP-ribosyl)transferase. J. Immunol..

[24] Riley J.P., Kulkarni A., Mehrotra P., Koh B., Perumal N.B., Kaplan M.H., Goenka S. (2013). Parp-14 binds specific DNA sequences to promote TH2 cell gene expression. PLoS One.

[25] Cho S.H., Goenka S., Henttinen T., Gudapati P., Reinikainen A., Eischen C.M., Lahesmaa R., Boothby M. (2009). PARP-14, a member of the b aggressive lymphoma family, transduces survival signals in primary b cells. Blood.

[26] Cho S.H., Ahn A.K., Bhargava P., Lee C.H., Eischen C.M., McGuinness O., Boothby M. (2011). Glycolytic rate and lymphomagenesis depend on PARP14, an adp ribosyltransferase of the B aggressive lymphoma (Bal) family. Proc. Natl. Acad. Sci. USA.

[27] Aguiar R.C., Yakushijin Y., Kharbanda S., Salgia R., Fletcher J.A., Shipp M.A. (2000). Bal is a novel risk-related gene in diffuse large B-cell lymphomas that enhances cellular migration. Blood.

[28] Camicia R., Bachmann S.B., Winkler H.C., Beer M., Tinguely M., Haralambieva E., Hassa P.O. (2013). Bal1/ARTD9 represses the anti-proliferative and pro-apoptotic ifngamma-STAT1-IRF1-p53 axis in diffuse large B-cell lymphoma. J. Cell. Sci..

[29] Bachmann S.B., Frommel S.C., Camicia R., Winkler H.C., Santoro R., Hassa P.O. (2014). DTX3l and ARTD9 inhibit IRF1 expression and mediate in cooperation with ARTD8 survival and proliferation of metastatic prostate cancer cells. Mol. Cancer.

[30] Verheugd P., Forst A.H., Milke L., Herzog N., Feijs K.L., Kremmer E., Kleine H., Luscher B. (2013). Regulation of NF-kappab signalling by the mono-ADP-ribosyltransferase artd10. Nat. Commun..

[31] Atasheva S., Frolova E.I., Frolov I. (2014). Interferon-stimulated poly(ADP-ribose) polymerases are potent inhibitors of cellular translation and virus replication. J. Virol..

[32] Kleine H., Herrmann A., Lamark T., Forst A.H., Verheugd P., Luscher-Firzlaff J., Lippok B., Feijs K.L., Herzog N., Kremmer E. (2012). Dynamic subcellular localization of the mono-ADP-ribosyltransferase ARTD10 and interaction with the ubiquitin receptor p62. Cell. Commun. Signal..

[33] Feijs K.L., Kleine H., Braczynski A., Forst A.H., Herzog N., Verheugd P., Linzen U., Kremmer E., Luscher B. (2013). ARTD10 substrate identification on protein microarrays: Regulation of gsk3beta by mono-ADP-ribosylation. Cell. Commun. Signal..

[34] Herzog N., Hartkamp J.D., Verheugd P., Treude F., Forst A.H., Feijs K.L., Lippok B.E., Kremmer E., Kleine H., Luscher B. (2013). Caspase-dependent cleavage of the mono-ADP-ribosyltransferase artd10 interferes with its pro-apoptotic function. Febs J..

[35] Welsby I., Hutin D., Gueydan C., Kruys V., Rongvaux A., Leo O. (2014). Parp12, an interferon-stimulated gene involved in the control of protein translation and inflammation. J. Biol. Chem..

[36] Bock F.J., Todorova T.T., Chang P. (2015). Rna regulation by poly(ADP-ribose) polymerases. Mol. Cell.

[37] Ma Q., Baldwin K.T., Renzelli A.J., McDaniel A., Dong L. (2001). Tcdd-inducible poly(ADP-ribose) polymerase: A novel response to 2,3,7,8-tetrachlorodibenzo-p-dioxin. Biochem. Biophys. Res. Commun..

[38] Ma Q. (2002). Induction and superinduction of 2,3,7,8-tetrachlorodibenzo-rho-dioxin-inducible poly(adp-ribose) polymerase: Role of the aryl hydrocarbon receptor/aryl hydrocarbon receptor nuclear translocator transcription activation domains and a labile transcription repressor. Arch. Biochem. Biophys..

[39] MacPherson L., Tamblyn L., Rajendra S., Bralha F., McPherson J.P., Matthews J. (2013). 2,3,7,8-Tetrachlorodibenzo-p-dioxin poly(ADP-ribose) polymerase (TIPARP, ARTD14) is a mono-ADP-ribosyltransferase and repressor of aryl hydrocarbon receptor transactivation. Nucleic Acids Res..

[40] Ahmed S., Bott D., Gomez A., Tamblyn L., Rasheed A., MacPherson L., Sugamori K.S., Cho T., Yang Y., Grant D.M. (2015). Loss of the mono-adp-ribosyltransferase, tiparp, increases sensitivity to dioxin-induced steatohepatitis and lethality. J. Biol. Chem..

[41] Atasheva S., Akhrymuk M., Frolova E.I., Frolov I. (2012). New parp gene with an anti-alphavirus function. J. Virol..

[42] Jwa M., Chang P. (2012). Parp16 is a tail-anchored endoplasmic reticulum protein required for the perk- and ire1alpha-mediated unfolded protein response. Nat. Cell. Biol..

[43] Tuncel H., Tanaka S., Oka S., Nakai S., Fukutomi R., Okamoto M., Ota T., Kaneko H., Tatsuka M., Shimamoto F. (2012). PARP6, a mono(ADP-ribosyl) transferase and a negative regulator of cell proliferation, is involved in colorectal cancer development. Int J. Oncol..

[44] Haigis M.C., Mostoslavsky R., Haigis K.M., Fahie K., Christodoulou D.C., Murphy A.J., Valenzuela D.M., Yancopoulos G.D., Karow M., Blander G. (2006). SIRT4 inhibits glutamate dehydrogenase and opposes the effects of calorie restriction in pancreatic beta cells. Cell.

[45] Jeong S.M., Xiao C., Finley L.W., Lahusen T., Souza A.L., Pierce K., Li Y.H., Wang X., Laurent G., German N.J. (2013). SIRT4 has tumor-suppressive activity and regulates the cellular metabolic response to DNA damage by inhibiting mitochondrial glutamine metabolism. Cancer Cell..

[46] Csibi A., Fendt S.M., Li C., Poulogiannis G., Choo A.Y., Chapski D.J., Jeong S.M., Dempsey J.M., Parkhitko A., Morrison T. (2013). The mtorc1 pathway stimulates glutamine metabolism and cell proliferation by repressing SIRT4. Cell.

[47] Mao Z., Hine C., Tian X., Van Meter M., Au M., Vaidya A., Seluanov A., Gorbunova V. (2011). SIRT6 promotes DNA repair under stress by activating parp1. Science.

[48] Mao Z., Tian X., Van Meter M., Ke Z., Gorbunova V., Seluanov A. (2012). Sirtuin 6 (SIRT6) rescues the decline of homologous recombination repair during replicative senescence. Proc. Natl. Acad. Sci. USA.

[49] McCord R.A., Michishita E., Hong T., Berber E., Boxer L.D., Kusumoto R., Guan S., Shi X., Gozani O., Burlingame A.L. (2009). SIRT6 stabilizes DNA-dependent protein kinase at chromatin for DNA double-strand break repair. Aging (Albany NY).

[50] Van Meter M., Kashyap M., Rezazadeh S., Geneva A.J., Morello T.D., Seluanov A., Gorbunova V. (2014). Sirt6 represses line1 retrotransposons by ribosylating kap1 but this repression fails with stress and age. Nat. Commun..

[51] Zhang J., Yin X.J., Xu C.J., Ning Y.X., Chen M., Zhang H., Chen S.F., Yao L.Q. (2015). The histone deacetylase SIRT6 inhibits ovarian cancer cell proliferation via down-regulation of notch 3 expression. Eur Rev. Med. Pharmacol. Sci..

[52] Fukuda T., Wada-Hiraike O., Oda K., Tanikawa M., Makii C., Inaba K., Miyasaka A., Miyamoto Y., Yano T., Maeda D. (2015). Putative tumor suppression function of SIRT6 in endometrial cancer. FEBS Lett..

[53] Zhang Z.G., Qin C.Y. (2014). Sirt6 suppresses hepatocellular carcinoma cell growth via inhibiting the extracellular signalregulated kinase signaling pathway. Mol. Med. Rep..

[54] Marquardt J.U., Fischer K., Baus K., Kashyap A., Ma S., Krupp M., Linke M., Teufel A., Zechner U., Strand D. (2013). Sirtuin-6-dependent genetic and epigenetic alterations are associated with poor clinical outcome in hepatocellular carcinoma patients. Hepatology.

[55] Sebastian C., Zwaans B.M., Silberman D.M., Gymrek M., Goren A., Zhong L., Ram O., Truelove J., Guimaraes A.R., Toiber D. (2012). The histone deacetylase SIRT6 is a tumor suppressor that controls cancer metabolism. Cell.

[56] Van Meter M., Mao Z., Gorbunova V., Seluanov A. (2011). Sirt6 overexpression induces massive apoptosis in cancer cells but not in normal cells. Cell. Cycle.

[57] Min L., Ji Y., Bakiri L., Qiu Z., Cen J., Chen X., Chen L., Scheuch H., Zheng H., Qin L. (2012). Liver cancer initiation is controlled by AP-1 through SIRT6-dependent inhibition of survivin. Nat. Cell. Biol..

[58] Liu Y., Xie Q.R., Wang B., Shao J., Zhang T., Liu T., Huang G., Xia W. (2013). Inhibition of SIRT6 in prostate cancer reduces cell viability and increases sensitivity to chemotherapeutics. Protein Cell..

[59] Ming M., Han W., Zhao B., Sundaresan N.R., Deng C.X., Gupta M.P., He Y.Y. (2014). SIRT6 promotes COX-2 expression and acts as an oncogene in skin cancer. Cancer Res..

[60] Han W.D., Mu Y.M., Lu X.C., Xu Z.M., Li X.J., Yu L., Song H.J., Li M., Lu J.M., Zhao Y.L. (2003). Up-regulation of LRP16 mrna by 17beta-estradiol through activation of estrogen receptor alpha (eralpha), but not erbeta, and promotion of human breast cancer MCF-7 cell proliferation: A preliminary report. Endocr. Relat. Cancer.

[61] Han W.D., Zhao Y.L., Meng Y.G., Zang L., Wu Z.Q., Li Q., Si Y.L., Huang K., Ba J.M., Morinaga H. (2007). Estrogenically regulated LRP16 interacts with estrogen receptor alpha and enhances the receptor’s transcriptional activity. Endocr. Relat. Cancer.

[62] Yang J., Zhao Y.L., Wu Z.Q., Si Y.L., Meng Y.G., Fu X.B., Mu Y.M., Han W.D. (2009). The single-macro domain protein LRP16 is an essential cofactor of androgen receptor. Endocr. Relat. Cancer.

[63] Li Y.Z., Zhao P., Han W.D. (2009). Clinicopathological significance of LRP16 protein in 336 gastric carcinoma patients. World J. Gastroenterol..

[64] Xi H.Q., Zhao P., Han W.D. (2010). Clinicopathological significance and prognostic value of LRP16 expression in colorectal carcinoma. World J. Gastroenterol..

[65] Mohseni M., Cidado J., Croessmann S., Cravero K., Cimino-Mathews A., Wong H.Y., Scharpf R., Zabransky D.J., Abukhdeir A.M., Garay J.P. (2014). Macrod2 overexpression mediates estrogen independent growth and tamoxifen resistance in breast cancers. Proc. Natl. Acad. Sci. USA.

[66] Sharifi R., Morra R., Appel C.D., Tallis M., Chioza B., Jankevicius G., Simpson M.A., Matic I., Ozkan E., Golia B. (2013). Deficiency of terminal ADP-ribose protein glycohydrolase TARG1/C6orf130 in neurodegenerative disease. Embo J..

[67] Liszt G., Ford E., Kurtev M., Guarente L. (2005). Mouse sir2 homolog sirt6 is a nuclear ADP-ribosyltransferase. J. Biol. Chem..

[68] Haigis M.C., Sinclair D.A. (2010). Mammalian sirtuins: Biological insights and disease relevance. Annu Rev. Pathol.

[69] Imai S., Guarente L. (2014). NAD^+^ and sirtuins in aging and disease. Trends Cell. Biol..

[70] Frye R.A. (1999). Characterization of five human cdnas with homology to the yeast SIR2 gene: SIR2-like proteins (sirtuins) metabolize nad and may have protein adp-ribosyltransferase activity. Biochem. Biophys Res. Commun..

[71] Tanny J.C., Dowd G.J., Huang J., Hilz H., Moazed D. (1999). An enzymatic activity in the yeast SIR2 protein that is essential for gene silencing. Cell.

[72] Daniels C.M., Ong S.E., Leung A.K. (2015). The promise of proteomics for the study of ADP-ribosylation. Mol. Cell.

[73] Haag F., Buck F. (2015). Identification and analysis of ADP-ribosylated proteins. Curr. Top. Microbiol. Immunol..

[74] Rosenthal F., Hottiger M.O. (2014). Identification of ADP-ribosylated peptides and adp-ribose acceptor sites. Front. Biosci. (Landmark Ed.).

[75] Otto H., Reche P.A., Bazan F., Dittmar K., Haag F., Koch-Nolte F. (2005). In silico characterization of the family of PARP-like poly(ADP-ribosyl)transferases (parts). BMC Genomics.

[76] Flick F., Luscher B. (2012). Regulation of sirtuin function by posttranslational modifications. Front. Pharmacol..

[77] Bai P. (2015). Biology of poly(ADP-ribose) polymerases: The factotums of cell maintenance. Mol. Cell.

[78] Feijs K.L., Forst A.H., Verheugd P., Luscher B. (2013). Macrodomain-containing proteins: Regulating new intracellular functions of mono(ADP-ribosyl)ation. Nat. Rev. Mol. Cell. Biol..

[79] Forst A.H., Karlberg T., Herzog N., Thorsell A.G., Gross A., Feijs K.L., Verheugd P., Kursula P., Nijmeijer B., Kremmer E. (2013). Recognition of mono-ADP-ribosylated ARTD10 substrates by ARTD8 macrodomains. Structure.

[80] Jankevicius G., Hassler M., Golia B., Rybin V., Zacharias M., Timinszky G., Ladurner A.G. (2013). A family of macrodomain proteins reverses cellular mono-ADP-ribosylation. Nat. Struct. Mol. Biol..

[81] Rosenthal F., Feijs K.L., Frugier E., Bonalli M., Forst A.H., Imhof R., Winkler H.C., Fischer D., Caflisch A., Hassa P.O. (2013). Macrodomain-containing proteins are new mono-ADP-ribosylhydrolases. Nat. Struct. Mol. Biol..

[82] Aguiar R.C., Takeyama K., He C., Kreinbrink K., Shipp M.A. (2005). B-aggressive lymphoma family proteins have unique domains that modulate transcription and exhibit poly(ADP-ribose) polymerase activity. J. Biol. Chem..

[83] Walford H.H., Doherty T.A. (2013). STAT6 and lung inflammation. JAKSTAT.

[84] Yu M., Schreek S., Cerni C., Schamberger C., Lesniewicz K., Poreba E., Vervoorts J., Walsemann G., Grotzinger J., Kremmer E. (2005). PARP-10, a novel myc-interacting protein with poly(ADP-ribose) polymerase activity, inhibits transformation. Oncogene.

[85] Kaufmann M., Feijs K.L., Luscher B. (2015). Function and regulation of the mono-ADP-ribosyltransferase artd10. Curr. Top. Microbiol. Immunol..

[86] Wu D., Pan W. (2010). Gsk3: A multifaceted kinase in WNT signaling. Trends Biochem. Sci..

[87] Beurel E., Michalek S.M., Jope R.S. (2010). Innate and adaptive immune responses regulated by glycogen synthase kinase-3 (GSK3). Trends Immunol..

[88] Chen Z.J. (2012). Ubiquitination in signaling to and activation of ikk. Immunol Rev..

[89] Hoesel B., Schmid J.A. (2013). The complexity of NF-kappab signaling in inflammation and cancer. Mol. Cancer.

[90] Gautheron J., Courtois G. (2010). “Without ub i am nothing”: Nemo as a multifunctional player in ubiquitin-mediated control of NF-kappab activation. Cell. Mol. Life Sci..

[91] Gardner B.M., Pincus D., Gotthardt K., Gallagher C.M., Walter P. (2013). Endoplasmic reticulum stress sensing in the unfolded protein response. Cold Spring Harb. Perspect Biol..

[92] Lee J., Ozcan U. (2014). Unfolded protein response signaling and metabolic diseases. J. Biol. Chem..

[93] Bertolotti A., Zhang Y., Hendershot L.M., Harding H.P., Ron D. (2000). Dynamic interaction of BiP and ER stress transducers in the unfolded-protein response. Nat. Cell. Biol..

[94] Hetz C. (2012). The unfolded protein response: Controlling cell fate decisions under ER stress and beyond. Nat. Rev. Mol. Cell. Biol..

[95] Di Paola S., Micaroni M., Di Tullio G., Buccione R., Di Girolamo M. (2012). PARP16/ARTD15 is a novel endoplasmic-reticulum-associated mono-ADP-ribosyltransferase that interacts with, and modifies karyopherin-SS1. PLoS ONE.

[96] Chambers J.E., Petrova K., Tomba G., Vendruscolo M., Ron D. (2012). ADP ribosylation adapts an ER chaperone response to short-term fluctuations in unfolded protein load. J. Cell. Biol..

[97] Fabrizio G., Di Paola S., Stilla A., Giannotta M., Ruggiero C., Menzel S., Koch-Nolte F., Sallese M., Di Girolamo M. (2015). Artc1-mediated ADP-ribosylation of GRP78/BiP: A new player in endoplasmic-reticulum stress responses. Cell. Mol. Life Sci..

[98] Nicolae C.M., Aho E.R., Vlahos A.H., Choe K.N., De S., Karras G.I., Moldovan G.L. (2014). The ADP-ribosyltransferase PARP10/ARTD10 interacts with proliferating cell nuclear antigen (PCNA) and is required for DNA damage tolerance. J. Biol. Chem..

[99] Moldovan G.L., Pfander B., Jentsch S. (2007). PCNA, the maestro of the replication fork. Cell.

[100] Nicolae C.M., Aho E.R., Choe K.N., Constantin D., Hu H.J., Lee D., Myung K., Moldovan G.L. (2015). A novel role for the mono-ADP-ribosyltransferase PARP14/artd8 in promoting homologous recombination and protecting against replication stress. Nucleic Acids Res..

[101] Beauharnois J.M., Bolivar B.E., Welch J.T. (2013). Sirtuin 6: A review of biological effects and potential therapeutic properties. Mol. Biosyst..

[102] Kugel S., Mostoslavsky R. (2014). Chromatin and beyond: The multitasking roles for SIRT6. Trends Biochem. Sci..

[103] Van Meter M., Mao Z., Gorbunova V., Seluanov A. (2011). Repairing split ends: SIRT6, mono-ADP ribosylation and DNA repair. Aging (Albany NY).

[104] Mostoslavsky R., Chua K.F., Lombard D.B., Pang W.W., Fischer M.R., Gellon L., Liu P., Mostoslavsky G., Franco S., Murphy M.M. (2006). Genomic instability and aging-like phenotype in the absence of mammalian SIRT6. Cell.

[105] Vyas S., Chang P. (2014). New parp targets for cancer therapy. Nat. Rev. Cancer.

[106] Dang C.V. (2012). Links between metabolism and cancer. Genes Dev..

[107] Jones R.G., Thompson C.B. (2009). Tumor suppressors and cell metabolism: A recipe for cancer growth. Genes Dev..

[108] Chiarugi A., Dolle C., Felici R., Ziegler M. (2012). The NAD metabolome—A key determinant of cancer cell biology. Nat. Rev. Cancer.

[109] Pollak N., Dolle C., Ziegler M. (2007). The power to reduce: Pyridine nucleotides—Small molecules with a multitude of functions. Biochem J..

[110] Scarpa E.S., Fabrizio G., Di Girolamo M. (2013). A role of intracellular mono-ADP-ribosylation in cancer biology. Febs J..

[111] Chou H.Y., Chou H.T., Lee S.C. (2006). CDK-dependent activation of poly(ADP-ribose) polymerase member 10 (parp10). J. Biol. Chem..

[112] Ben-Neriah Y., Karin M. (2011). Inflammation meets cancer, with NF-kappab as the matchmaker. Nat. Immunol..

[113] Raval-Fernandes S., Kickhoefer V.A., Kitchen C., Rome L.H. (2005). Increased susceptibility of vault poly(ADP-ribose) polymerase-deficient mice to carcinogen-induced tumorigenesis. Cancer Res..

[114] Huang G., Cui F., Yu F., Lu H., Zhang M., Tang H., Peng Z. (2015). Sirtuin-4 (sirt4) is downregulated and associated with some clinicopathological features in gastric adenocarcinoma. Biomed. Pharmacother..

[115] Miyo M., Yamamoto H., Konno M., Colvin H., Nishida N., Koseki J., Kawamoto K., Ogawa H., Hamabe A., Uemura M. (2015). Tumour-suppressive function of SIRT4 in human colorectal cancer. Br. J. Cancer.

[116] Blaveri E., Simko J.P., Korkola J.E., Brewer J.L., Baehner F., Mehta K., Devries S., Koppie T., Pejavar S., Carroll P. (2005). Bladder cancer outcome and subtype classification by gene expression. Clin. Cancer Res..

[117] Zhao Y.L., Han W.D., Li Q., Mu Y.M., Lu X.C., Yu L., Song H.J., Li X., Lu J.M., Pan C.Y. (2005). Mechanism of transcriptional regulation of LRP16 gene expression by 17-beta estradiol in mcf-7 human breast cancer cells. J. Mol. Endocrinol..

[118] Liang J., Shang Y. (2013). Estrogen and cancer. Annu Rev. Physiol..

[119] Meng Y.G., Han W.D., Zhao Y.L., Huang K., Si Y.L., Wu Z.Q., Mu Y.M. (2007). Induction of the LRP16 gene by estrogen promotes the invasive growth of ishikawa human endometrial cancer cells through the downregulation of E-cadherin. Cell. Res..

[120] Forbes S.A., Beare D., Gunasekaran P., Leung K., Bindal N., Boutselakis H., Ding M., Bamford S., Cole C., Ward S. (2015). Cosmic: Exploring the world’s knowledge of somatic mutations in human cancer. Nucleic Acids Res..

[121] Akira S., Uematsu S., Takeuchi O. (2006). Pathogen recognition and innate immunity. Cell.

[122] Trocoli A., Djavaheri-Mergny M. (2011). The complex interplay between autophagy and NF-kappab signaling pathways in cancer cells. Am. J. Cancer Res..

[123] Daugherty M.D., Young J.M., Kerns J.A., Malik H.S. (2014). Rapid evolution of PARP genes suggests a broad role for ADP-ribosylation in host-virus conflicts. PLoS Genet..

[124] Bick M.J., Carroll J.W., Gao G., Goff S.P., Rice C.M., MacDonald M.R. (2003). Expression of the zinc-finger antiviral protein inhibits alphavirus replication. J. Virol..

[125] Gao G., Guo X., Goff S.P. (2002). Inhibition of retroviral RNA production by ZAP, a CCCH-type zinc finger protein. Science.

[126] Mao R., Nie H., Cai D., Zhang J., Liu H., Yan R., Cuconati A., Block T.M., Guo J.T., Guo H. (2013). Inhibition of hepatitis B virus replication by the host zinc finger antiviral protein. PLoS Pathog.

[127] Muller S., Moller P., Bick M.J., Wurr S., Becker S., Gunther S., Kummerer B.M. (2007). Inhibition of filovirus replication by the zinc finger antiviral protein. J. Virol..

[128] Zhu Y., Chen G., Lv F., Wang X., Ji X., Xu Y., Sun J., Wu L., Zheng Y.T., Gao G. (2011). Zinc-finger antiviral protein inhibits HIV-1 infection by selectively targeting multiply spliced viral mrnas for degradation. Proc. Natl. Acad. Sci. USA.

[129] Chen S., Xu Y., Zhang K., Wang X., Sun J., Gao G., Liu Y. (2012). Structure of N-terminal domain of ZAP indicates how a zinc-finger protein recognizes complex rna. Nat. Struct. Mol. Biol..

[130] Guo X., Carroll J.W., Macdonald M.R., Goff S.P., Gao G. (2004). The zinc finger antiviral protein directly binds to specific viral mrnas through the CCCH zinc finger motifs. J. Virol..

[131] Jeong M.S., Kim E.J., Jang S.B. (2010). Expression and RNA-binding of human zinc-finger antiviral protein. Biochem. Biophys. Res. Commun..

[132] Guo X., Ma J., Sun J., Gao G. (2007). The zinc-finger antiviral protein recruits the RNA processing exosome to degrade the target mrna. Proc. Natl. Acad. Sci. USA.

[133] Glasker S., Toller M., Kummerer B.M. (2014). The alternate triad motif of the poly(ADP-ribose) polymerase-like domain of the human zinc finger antiviral protein is essential for its antiviral activity. J. Gen. Virol..

[134] Buchan J.R., Parker R. (2009). Eukaryotic stress granules: The INS and outs of translation. Mol. Cell.

[135] Leung A., Todorova T., Ando Y., Chang P. (2012). Poly(ADP-ribose) regulates post-transcriptional gene regulation in the cytoplasm. RNA Biol..

[136] Beckham C.J., Parker R. (2008). P bodies, stress granules, and viral life cycles. Cell Host Microbe.

[137] Onomoto K., Yoneyama M., Fung G., Kato H., Fujita T. (2014). Antiviral innate immunity and stress granule responses. Trends Immunol..

[138] Egloff M.P., Malet H., Putics A., Heinonen M., Dutartre H., Frangeul A., Gruez A., Campanacci V., Cambillau C., Ziebuhr J. (2006). Structural and functional basis for ADP-ribose and poly(ADP-ribose) binding by viral macro domains. J. Virol..

[139] Neuvonen M., Ahola T. (2009). Differential activities of cellular and viral Macro domain proteins in binding of ADP-ribose metabolites. J. Mol. Biol..

[140] Saikatendu K.S., Joseph J.S., Subramanian V., Clayton T., Griffith M., Moy K., Velasquez J., Neuman B.W., Buchmeier M.J., Stevens R.C. (2005). Structural basis of severe acute respiratory syndrome coronavirus ADP-ribose-1′′-phosphate dephosphorylation by a conserved domain of NSP3. Structure.

[141] Eriksson K.K., Cervantes-Barragan L., Ludewig B., Thiel V. (2008). Mouse hepatitis virus liver pathology is dependent on ADP-ribose-1′′-phosphatase, a viral function conserved in the alpha-like supergroup. J. Virol..

[142] Park E., Griffin D.E. (2009). The NSP3 macro domain is important for sindbis virus replication in neurons and neurovirulence in mice. Virology.

[143] Kuri T., Eriksson K.K., Putics A., Zust R., Snijder E.J., Davidson A.D., Siddell S.G., Thiel V., Ziebuhr J., Weber F. (2011). The ADP-ribose-1′′-monophosphatase domains of severe acute respiratory syndrome coronavirus and human coronavirus 229e mediate resistance to antiviral interferon responses. J. Gen. Virol..

[144] Rack J.G., Morra R., Barkauskaite E., Kraehenbuehl R., Ariza A., Qu Y., Ortmayer M., Leidecker O., Cameron D.R., Matic I. (2015). Identification of a class of protein ADP-ribosylating sirtuins in microbial pathogens. Mol. Cell.

[145] Holbourn K.P., Shone C.C., Acharya K.R. (2006). A family of killer toxins. Exploring the mechanism of ADP-ribosylating toxins. FEBS J..

[146] Deng Q., Barbieri J.T. (2008). Molecular mechanisms of the cytotoxicity of ADP-ribosylating toxins. Annu. Rev. Microbiol..

